# Bacterial Diversity and Vertical Distribution Patterns in Sandy Sediments: A Study on the Bacterial Community Structure Based on Environmental Factors in Tributaries of the Yangtze River

**DOI:** 10.3390/microorganisms12061178

**Published:** 2024-06-11

**Authors:** Tian Zhang, Weibo Wang, Yifei Leng, Yu Huang, Wen Xiong, Fengyi Chang

**Affiliations:** 1 Department of Civil Engineering, Architecture and Environment, Hubei University of Technology, Wuhan 430068, China; ztcxzks@163.com (T.Z.); yifeileng@126.com (Y.L.); huangyu@hbut.edu.cn (Y.H.); wenx2217@163.com (W.X.); 2 Wuhan Botanical Garden of the Chinese Academy of Sciences, Wuhan 430074, China; wangweibo@wbgcas.cn; 3 Innovation Demonstration Base of Ecological Environment Geotechnical and Ecological Restoration of Rivers and Lakes, Hubei University of Technology, Wuhan 430068, China; 4 Key Laboratory of Intelligent Health Perception and Ecological Restoration of Rivers and Lakes, Ministry of Education, Hubei University of Technology, Wuhan 430068, China

**Keywords:** Yangtze river basin, sandy sediment, bacterial diversity, vertical distribution

## Abstract

Bacterial diversity and its distribution characteristics in sediments are critical to understanding and revealing biogeochemical cycles in sediments. However, little is known about the relationship between biogeochemistry processes and vertical spatial distribution of bacterial communities in sandy sediments. In this study, we used fluorescence quantitative PCR, high-throughput sequencing technology and statistical analysis to explore the vertical distribution pattern of bacterial community diversity and its influencing factors in sandy sediments of the Yangtze River Basin. The aim is to enrich the understanding of the ecological characteristics and functions of bacteria in river ecosystems. The results showed that both sediment bacterial abundance and diversity showed a gradual decrease from surface to bottom in the vertical distribution. The main environmental factors that influenced the bacterial distribution pattern were pore water dissolved oxygen (DO), total nitrogen (TN) concentration and sediment nitrogen (N) content. The dominant bacterial species, *Massilia* and *Flavobacterium*, are suitable for growth and reproduction in high oxygen and nutrient-richer environments, while *Limnobacter* prefers low oxygen or anaerobic conditions. The vertical distribution pattern of bacteria and its influencing factors in river sandy sediment found in this study differ from the results in mud sediment, which may be related to the larger granular gap between sandy sediment and the lower content of organic matter. The findings of this study further our understanding of the distribution patterns and ecological preferences of microbial communities in river sediments, providing insights into how these communities may adapt to varying environmental conditions.

## 1. Introduction

Sandy sediments play a crucial role in river ecosystems, serving as a dynamic site for physical, chemical and microbiological processes [1]. Microbes within these sediments are key players in driving biogeochemical cycles and are vital indicators of ecosystem health and functionality. Notably, the surface layers of sediments are typically inhabited by *Actinomycetes* and *Cyanobacteria*, two genera that play distinct yet complementary roles in nutrient cycling. *Actinomycetes*, known for their metabolic versatility, actively participate in the cycling of elements such as nitrogen and sulfur, contributing significantly to the biogeochemical dynamics of the ecosystem. Meanwhile, *Cyanobacteria* contribute to primary productivity in river ecosystems through photosynthesis, facilitating carbon cycling and supporting the base of the food web. Deeper within the sediments, anaerobic ammonia-oxidizing bacteria (*Anammox*) emerge as crucial players in the nitrogen cycle, mediating the conversion of ammonium to nitrogen gas under anoxic conditions. The rich aggregation of organic matter and nutrients in sediment surfaces makes this complex interplay of microorganisms, underscoring the critical importance of sandy sediments in maintaining the vitality and resilience of riverine environments [2,3].

With the increasing research in microbiology, especially bacteriology, understanding the spatial distribution of bacteriology communities in sandy sediments has emerged as a significant area of study. Previous studies have provided valuable insights into the vertical distribution of microbial communities in aquatic sediments, while existing research predominantly focuses on lakes and reservoirs, particularly in horizontal spatial distribution patterns. For instance, Allen et al. [4] demonstrated the impact of oxygen availability on microbial diversity in lake sediments, while Wyness et al. [5] highlighted the role of nutrient gradients in shaping community composition in estuarine environments. However, these studies have several limitations that our research aims to address. Firstly, much of the existing literature has focused on lake or marine sediments, with relatively few studies examining the unique characteristics of riverine sandy sediments, which are subject to different environmental pressures and dynamics [6]. Secondly, while some studies have considered the influence of individual factors such as oxygen or nutrients, a comprehensive assessment of multiple interacting factors across different depths is lacking. Especially compared with other water environments, river sediments are usually coarser and show obvious stratification. The microbial diversity is more complex and has a faster metabolic rate [7]. Consequently, conducting such studies can further enrich the understanding of microbial diversity in fluvial sandy sediments.

The vertical spatial distribution pattern of bacterial communities in sandy sediments is a complex phenomenon influenced by a combination of factors, including sediment physical structure, chemical conditions, biological activity and geochemical processes. The physical structure of the sediment, particularly variations in grain size and porosity, plays a significant role in shaping the vertical distribution of microorganisms. Larger sand grains provide more voids, allowing for greater oxygen and nutrient penetration, which in turn creates areas of high bacterial activity in the surface layer. In contrast, decreasing porosity with depth restricts oxygen penetration, leading to changes in bacterial community structure [8]. Additionally, factors such as pH, redox potential, organic matter content and nutrient salt concentration also exert a profound influence on the vertical distribution pattern of microorganisms. For example, the high permeability of the water flow allows for rapid passage through the sediment layers, carrying large amounts of external organic matter and nutrients into the sediments. Microbial biochemical processes on sediment surfaces can also aggregate organic matter and nutrients. Fertilizers and pesticides used in agricultural activities enter rivers with surface runoff, increasing the nutrient content of the sediment surface. Consequently, sediment surfaces tend to be rich in organic matter and nutrients, supporting high levels of bacterial biomass and diversity, whereas deeper sediments may exhibit reduced bacterial abundance and diversity due to decomposition of organic matter and nutrient depletion [9].

The activities of benthic animals and other organisms can also impact the physical structure of the sediment, promoting mixing and cycling of organic matter and nutrients, and thereby affecting the distribution and activity of the bacterial community [10]. Furthermore, geochemical processes, including redox reactions, mineralization of organic matter, and nutrient cycling, have a significant impact on the vertical distribution patterns of microorganisms [11]. Aerobic processes dominate in surface sediments, while anaerobic processes, such as sulfate reduction and methanogenesis, may be more prevalent in deeper sediments, influencing the type of metabolism and spatial distribution of microorganisms. Recent studies have investigated the vertical distribution of bacterial diversity in sandy sediments of different rivers, highlighting the importance of environmental factors such as oxygen concentration, nutrient availability, and pollutant concentration. Wang et al. found that the bacterial diversity of sandy sediments varied significantly with environmental factors such as oxygen concentration, nutrient availability and pollutant concentration from the surface to the bottom [12]. Liu et al. concluded that TOC and TP were the main environmental factors affecting the vertical distribution of bacterial communities in Qiantang River sediments [13].

In general, the introduction of nutrients can alter the chemical composition of sediments, subsequently affecting microbial community structure and function. However, while recognizing this overarching influence, the specific mechanisms driving the vertical distribution of microorganisms within riverine sandy sediments remain veiled in mystery. Considering the unique environmental characteristics and the paramount importance of microbial assemblages in orchestrating biogeochemical cycles, we advance a hypothesis that the stratification of microbial diversity within the sandy sediments of the Yangtze River Basin will manifest distinctive patterns, intricately shaped by prevailing environmental gradients.

## 2. Materials and Methods

The Yangtze River is the third largest river in the world and the largest in China, and its catchment area is vast and covers a number of provinces and regions. Vegetation cover in the basin is generally “high in the center and low in the east and west”. The Yangtze River Basin has complex and varied climatic conditions, ranging from humid subtropical climates to cold highland climates. Such diverse climatic conditions support rich biodiversity and different ecosystem types in the region. Higher water temperatures in the summer and lower temperatures in the winter have a direct impact on the survival and reproduction of organisms in the watershed [14]. The eutrophication ratio of lakes and reservoirs in the middle reaches of the Yangtze River Basin increased from 31.3% in 2009 to 42.7% in 2018, and the nutrient levels of reservoirs are rapidly evolving from mesotrophic to mildly eutrophic [15].

As one of China’s most vital water systems, the stability of its ecosystem is closely monitored by stakeholders across various sectors. To better understand the vertical distribution patterns of microorganisms in riverine sandy sediments and the factors influencing them, this study focused on the sandy sediments in dry tributaries of the Yangtze River. We selected three sampling sites: Baihushan (Yangtze River), Huangzhuang (Han River), and Shengmi (Gan River) were earmarked to assess the physicochemical indices of the sediments and their pore water at varying depths. Concurrently, bacterial DNA was extracted from these sediment samples to examine the ecosystem’s robustness and resilience across different depth gradients. Techniques such as fluorescence quantitative PCR and 16S rRNA sequencing were employed, with the bacterial V4-1 gene serving as a biomarker, to explore the distribution patterns of microorganisms in sandy sediments at various depths and to analyze their correlation with a spectrum of environmental factors. The objective is to elucidate the vertical distribution characteristics of microorganisms in the sandy sediments of the Yangtze River Basin, to enhance our understanding of the adaptability of bacterial communities under diverse environmental conditions, their responses to ecological changes, and to offer theoretical guidance for the conservation and restoration of riverine ecosystems.

### 2.1. Sample Collection

**Figure 1:**
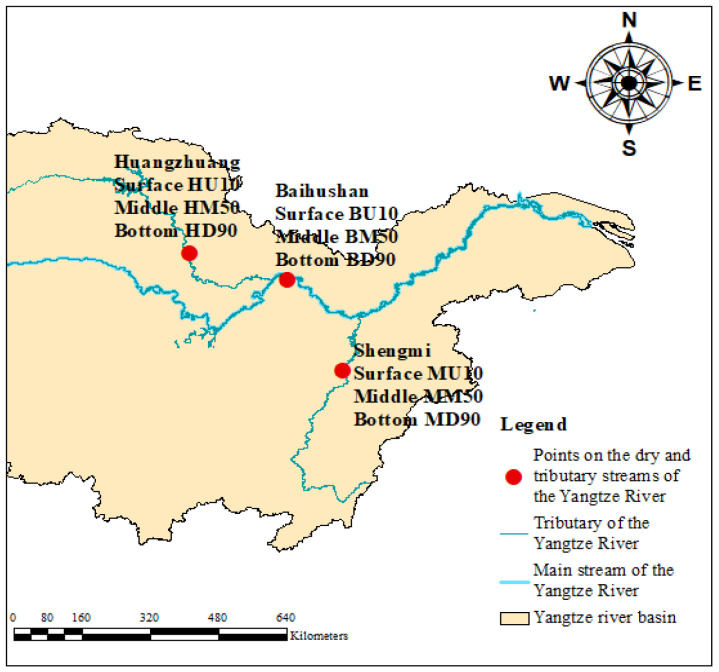
Baihushan, Huangzhuang and Shengmi sampling sites.

In this study, the sandy sediments of the main tributaries of the Yangtze River were taken as the object, and one sampling point each was set up in Baihushan (E 114.61°, N 30.57°), Huangzhuang (E 112.55°, N 31.18°) and Shengmi (E 115.78°, N 28.53°), and the sampling points are shown in Figure 1, among which A, B, C and D are selected at intervals of 100m along the riverbanks at the sample points in Baihushan and Huangzhuang sample points, the use of 10 cm diameter gravity column sediment collector to collect surface (10 cm), middle (50 cm) and bottom (90 cm) sediment samples of 500 g, the use of HR-Peeper pore water collector to collect the surface (10 cm), middle (50 cm) and bottom (90 cm) pore water samples of 500 mL. Two sample points, A and B, were selected at 200 m intervals from the Shengmi sample points, and sediment and pore water samples were collected by the same method. The above pore water sample containers were moistened in advance, 1ml of sulfuric acid solution was added to the samples, and the sediment samples were put into sterile plastic self-sealing bags and placed together in a 4 °C car refrigerator, and then transferred to the laboratory refrigerator for preservation.

### 2.2. Measurement of Physical and Chemical Indicators

Sediment samples were analyzed for total nitrogen (TN), total organic carbon (TOC), and total carbon (TC) content using a solid sample carbon analyzer (soli TOC cube). Particle size was determined using a laser particle size and shape analyzer (Mastersizer 3000). Pore water samples were analyzed for dissolved oxygen (DO) on-site using an automatic dissolved oxygen meter (WP-82Y). Total nitrogen (TN) in the pore water was measured with a TOC analyzer (Vario TOC). Other water quality parameters were determined in accordance with the protocols outlined in “Methods of Analysis for Water and Wastewater Monitoring (Fourth Edition) [16]”. Ammonium nitrogen (NH_4_^+^-N) was measured using Nessler’s reagent spectrophotometry, nitrate nitrogen (NO_3_-N) by phenol disulfonic acid spectrophotometry, and chemical oxygen demand (COD_Mn_) using the acidic permanganate method.

### 2.3. Bacterial Diversity Measurement

Enrichment of bacterial DNA with reference to the method reported by Li et al. [17]. Approximately 50 g of thoroughly dried sediment samples were placed into a 100 mL beaker, and distilled water was added until it reached no more than two-thirds of the sample volume. The mixed samples were then transferred into an ultrasonic cleaner, ensuring that the water level in the cleaner did not exceed the liquid level in the beaker. The samples were subjected to ultrasonic agitation for 5 min. Following this, the turbid liquid was decanted into pre-weighed 50 mL centrifuge tubes. The tubes were then placed into a centrifuge set at 5 °C with a speed of 5000 rpm. After centrifugation, the supernatant was discarded. This process was repeated 7 to 8 times, with distilled water added each time until the color of the suspension notably lightened following ultrasonic agitation. The processed samples were then placed into a freeze dryer. Once drying was complete, the samples were weighed to calculate the mass of the extracted material.

### 2.4. Fluorescence Quantitative qPCR Assay

The abundance of bacterial 16S rRNA gene V4 region was quantified using qPCR on a BioER 9600 Plus instrument (BioER Technology Co., Hangzhou, China), with the primers 515F and 806R, which correspond to the sequences GTGCCAGCMGCCGCGGTAA and GGACTACHVGGGTWTCTAAT, respectively. This approach targets a specific hypervariable region of the 16S rRNA gene that is commonly used for bacterial community analysis. The specific methodology was as follows: A 5 μL aliquot of DNA stock solution was taken and diluted with 20 μL of nuclease-free water to achieve a 5-fold dilution. The diluted sample was then amplified using the designated primers. The PCR products were subjected to agarose gel electrophoresis, and the corresponding DNA bands were excised from the gel to recover the target DNA fragment. This fragment was ligated into a Pgem-T Easy Vector (3015 bp)(Biolabs Technology Co., Beijing, China) and subsequently transformed into competent DH5a *Escherichia coli* cells. The cells were plated on a Petri dish containing benzylpenicillin and incubated overnight to culture monoclonal strains. These strains were verified using qPCR, followed by expanded culturing, and then plasmid DNA was extracted.

To convert the vector concentration to copies per microliter (copies/μL), the following formula was used:

Copies/μL = [6.02 × 10^23^ × (ng/uL) × 10^−9^]/fragment length × 660.

Based on this concentration, plasmid DNA was diluted to prepare standard curves with concentrations of 10^10^, 10^9^, 10^8^, 10^7^, 10^6^, and 10^5^ copies/uL. For fluorescence quantification, 2× ChamQ SYBR Color qPCR Master Mix was utilized (High ROX Premixed, Nanjing Novozymes Biotechnology Co., Ltd., Nanjing, China).

### 2.5. Bacterial 16s rRNA Sequencing

Genomic DNA was extracted from each type of sample following the guidelines provided with the respective DNA extraction kits. The integrity and purity of the DNA were assessed through 1% agarose gel electrophoresis, and the concentration and purity were measured using a NanoDrop One spectrophotometer (Thermo Fisher Scientific, Wilmington, DE, USA). PCR amplification was performed using the genomic DNA as a template, employing primers with barcodes and PremixTaq (TaKaRa) (TaKaRa Biotechnology (Dalian) Co., Ltd., Dalian, China) in accordance with the selected sequencing region. PCR product concentrations were evaluated using GeneTools Analysis Software (Version 4.03.05.0, SynGene (Syngene International Ltd., Bangalore, India)), and the volume of each sample required for equal mass pooling was calculated. Mixed PCR products were purified using the E.Z.N.A.^®^ Gel Extraction Kit (VWR International, Lutterworth, UK), and the target DNA fragments were eluted with TE buffer (Thermo Fisher Scientific, Wilmington, DE, USA).

Library construction for sequencing was conducted following the standard protocol of the NEBNext^®^ Ultra™ DNA Library Prep Kit for Illumina^®^ (Illumina Inc., San Diego, CA, USA). Upon completion, sequencing was carried out on high-throughput platforms such as HiSeq or MiSeq with paired-end reads. The raw image data files generated from sequencing were processed through base calling, converting them into raw reads, which included the sequence data and their corresponding quality information. The bacterial sequences obtained from this study have been deposited in the NCBI under accession numbers PRJNA1117588.

### 2.6. Data Processing and Analysis

The physicochemical index data for the pore water and sediments from the sampling sites, as well as redundancy analysis between dominant microorganisms and environmental factors, were processed using Origin 2021b Pro software. Bacterial diversity and environmental factors were analyzed via ANOVA and correlation analysis with IBM SPSS Statistics 27 software. Graphs and visual data representations were also generated using Origin 2021b Pro software.

## 3. Results

### 3.1. Changes in Physical and Chemical Indicators at Various Depths

**Figure 2:**
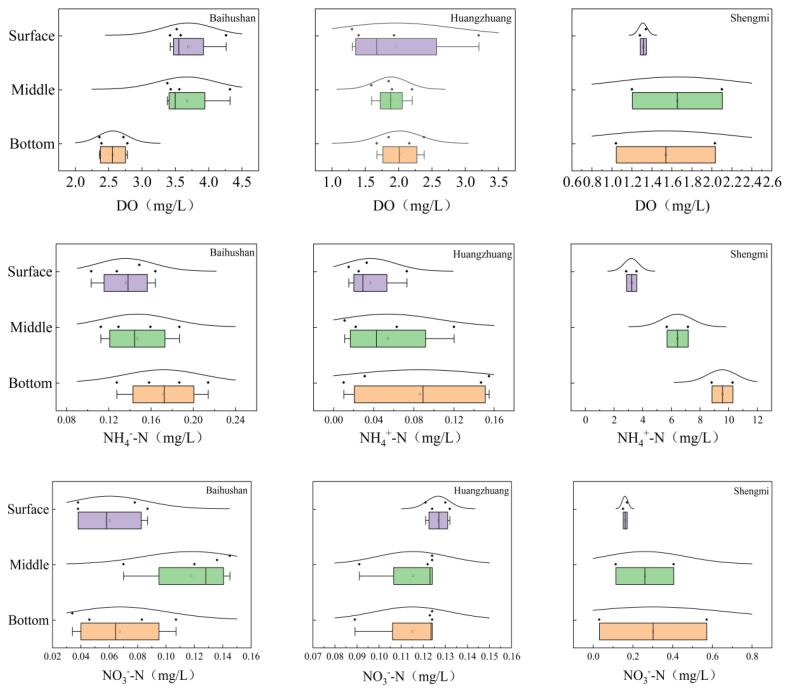

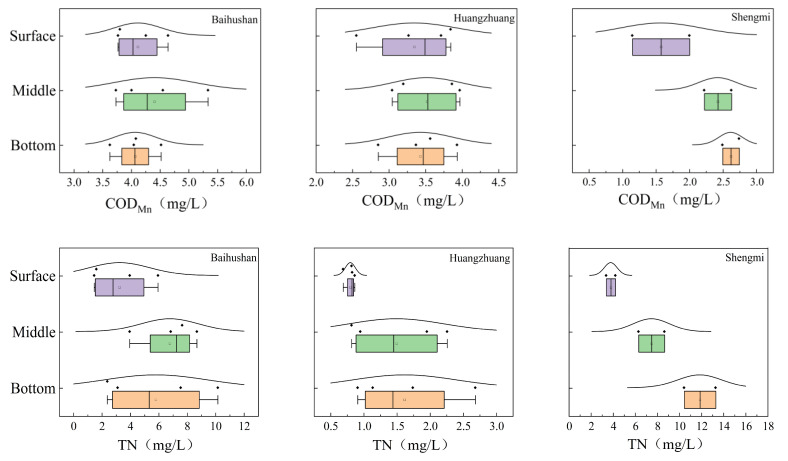
Variation of physicochemical of interstitial water at different depths. Purple boxplots represent the surface samples, green boxplots represent the middle samples, and orange boxplots represent the bottom samples. Each dot represents a single data point. The curves represent the distribution characteristics of the sample point data.

As shown in Figure 2, based on statistical analysis: The average DO concentration in the interstitial water of the sample site at Baihushan is significantly higher in the surface and middle layers than in the bottom layer (*p* < 0.05). Average concentrations of NH_4_^+^-N, NO_3_^−^-N, COD_Mn_ and TN were all lowest in the surface layer, with a tendency to increase gradually with increasing depth. In addition, the average NO_3_^−^-N concentration was considerably higher in the middle layer than in the surface and bottom layers (*p* < 0.05). There were no significant differences in the concentrations of other physicochemical indicators in the vertical direction (*p* > 0.05). The average concentrations of DO, NH_4_^+^-N and TN in the interstitial water at Huangzhuang sample site showed a relatively consistent trend in general, all of which showed that the bottom layer > middle layer > surface layer. However, no significant differences in NO_3_^−^-N and COD_Mn_ concentrations were detected between sedimentary layers (*p* > 0.05). The physicochemical indicators of interstitial water in the Shengmi sample sites can be observed as a relatively obvious trend variation. The average concentration of each indicator gradually increased from the surface to the bottom. Among them, DO, NH_4_^+^-N and TN varied more significantly, and the average concentration in the surface layer was significantly lower than that in the middle and bottom layers (*p* < 0.05).

**Figure 3:**
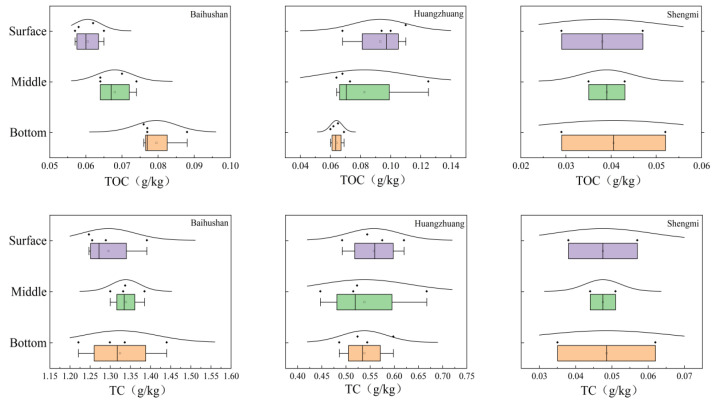

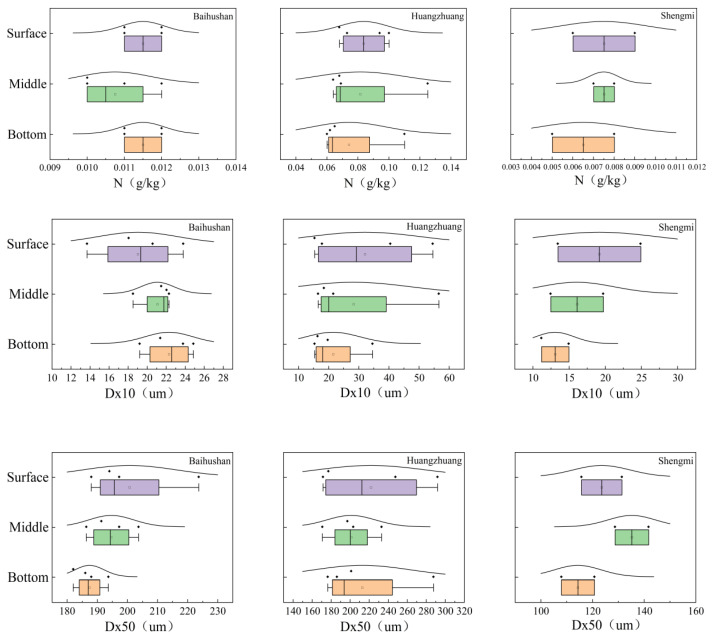
Variation of physicochemical indicators in sediments at different depths. Purple boxplots represent the surface samples, green boxplots represent the middle samples, and orange boxplots represent the bottom samples. Each dot represents a single data point. The curves represent the distribution characteristics of the sample point data.

The results obtained from the statistical analysis of sediment physicochemical indicators are shown in Figure 3. The sediment TOC, TC and N content and Dx10 of the Baihushan sample site showed a consistent trend from the surface to the bottom, all being lowest in the surface layer and gradually increased with the depth. Among them, the TOC content of the bottom layer was significantly higher than that of the surface and middle layers (*p* < 0.05). On the other hand, sediment Dx50 is shown to be the largest in the surface layer and the smallest in the bottom layer. The physiochemical indexes of each sediment in Huangzhuang sample site showed obvious trends, with the characteristics of surface layer > middle layer > bottom layer, but the differences between the sediment layers were not significant (*p* > 0.05). There was no significant variability in TOC, TC and N contents among sedimentary layers in the Shengmi sample sites (*p* > 0.05). However, the trends of Dx10 and Dx50 have similar features to those of the Huangzhuang sample points, both of which are minimized at the bottom.

### 3.2. Variations in Gene Abundance at Different Depths in Bacterial

**Figure 4:**
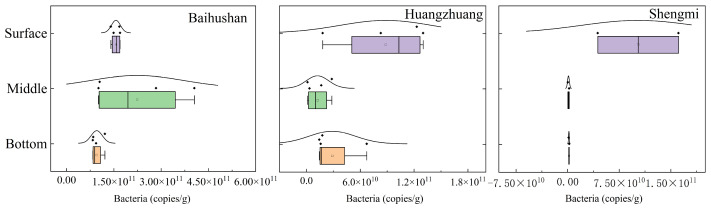
Variation in bacterial V4-1 gene abundance. Purple boxplots represent the surface samples, green boxplots represent the middle samples, and orange boxplots represent the bottom samples. Each dot represents a single data point. The curves represent the distribution characteristics of the sample point data.

A relatively significant variation in the vertical distribution of bacterial V4-1 gene abundance can be noted in Figure 4. The average values of bacterial abundance from the surface layer to the bottom layer of the sample site at Baihushan were 1.56 × 10^11^, 2.24 × 10^11^, and 9.59 × 10^10^copies/g, respectively, with a general trend of gradual decrease. On the other hand, the abundance averages of the Huangzhuang and Shengmi sample sites were 8.85 × 10^10^, 1.23 × 10^10^, 2.88 × 10^10^copies/g and 1.03 × 10^11^, 1.18 × 10^9^, 1.85 × 10^9^copies/g, respectively. The two samples shared similar abundance profiles, with both surface abundance being significantly larger than the mid- and bottom layer (*p* < 0.05). Trends in sediment bacterial abundance may be related to differences in nutrients in different sediment layers.

### 3.3. Bacterial Community Structure Analysis

Statistics on the taxonomic levels of sediment microorganisms at the phylum level and genus level in the sample sites, and calculation of the relative abundance of each taxon. At the same time, taxa with relative abundance above 1% were selected to analyze the dominant microorganisms with the highest abundance in each sample site.

**Figure 5:**
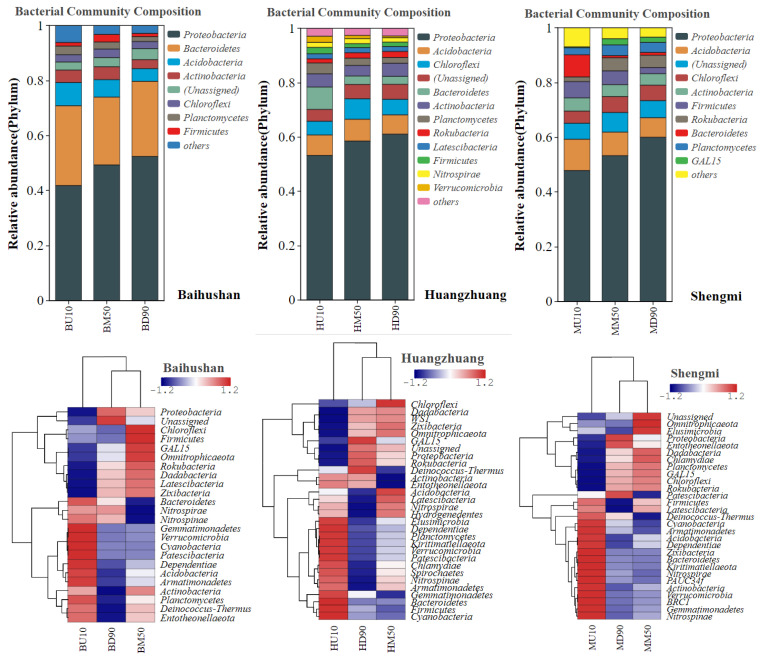
Bacterial phylum level community composition and abundance clustering analysis. BU10, BD90, BM50 represent samples from the surface layer (10 cm), bottom layer (90 cm) and middle layer (50 cm) of Baishan sediment, respectively. HU10, HD90, HM50 represent samples from the surface layer (10 cm), bottom layer (90 cm) and middle layer (50 cm) of Huangzhuang, respectively. MU10, MD90, MM50 represent samples from the surface layer (10 cm), bottom layer (90 cm) and middle layer (50 cm) of Shengmi, respectively.

Figure 5 and Table 1 showsshows microorganisms from the Baihushan sample site at the phylum level. *Proteobacteria* and *Bacteroidetes* were significantly dominant species, accounting for 41.8%, 49.3%, 52.4% and 29%, 24.7%, 27.3% in the surface, middle and bottom layers, respectively. At the Huangzhuang and Shengmi sample sites, *Proteobacteria* still had a significant advantage, accounting for 53.2%, 58.5%, 61% and 47.8%, 52.3%, 60% in the surface layer, middle layer and bottom layer, respectively.

**Figure 6:**
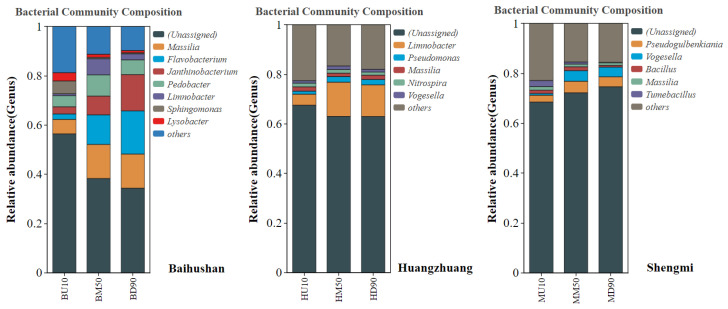

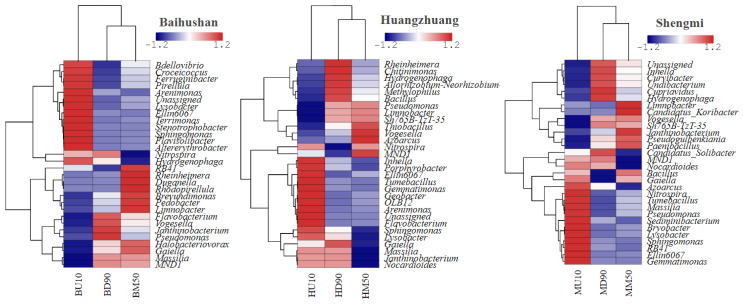
Bacterial genus level community composition and abundance clustering analysis. BU10, BD90, BM50 represent samples from the surface layer (10 cm), bottom layer (90 cm) and middle layer (50 cm) of Baishan sediment, respectively. HU10, HD90, HM50 represent samples from the surface layer (10 cm), bottom layer (90 cm) and middle layer (50 cm) of Huangzhuang, respectively. MU10, MD90, MM50 represent samples from the surface layer (10 cm), bottom layer (90 cm) and middle layer (50 cm) of Shengmi, respectively.

The community composition of sediment microorganisms at the genus level is shown in Figure 6. and Table 2. From surface to bottom layer, 56.4%, 38.3% and 34.3% of bacterial species could not be identified at the Baihushan sample site, respectively. In addition, the dominant species in the surface layer were *Massilia* (5.9%), *Sphingomonas* (5.2%) and *Pedobacter* (4.5%); *Massilia* (13.8%), *Flavobacterium* (12%) and *Pedobacter* (8.6%) were predominantly dominant in the middle layer. In the bottom layer, *Flavobacterium* (17.5%), *Janthinobacterium* (14.7%) and *Massilia* (13.9%) were more abundant. *Massilia* was the significantly dominant species at the sample site. The percentage of unidentified bacterial species was 67.5%, 63%, 63.1% and 68.5%, 72.2%, 74.6% at the Huangzhuang and Shengmi sample sites, respectively. Moreover, *Limnobacter* was significantly dominant at the Huangzhuang sample site with a percentage of 4.4%, 13.8%, and 12.8%, respectively, whereas *Pseudogulbenkiania* dominated at the Shengmi sample sites, accounting for 2.7%, 4.6%, and 4%, respectively.

### 3.4. Alpha Diversity Analysis

**Figure 7:**
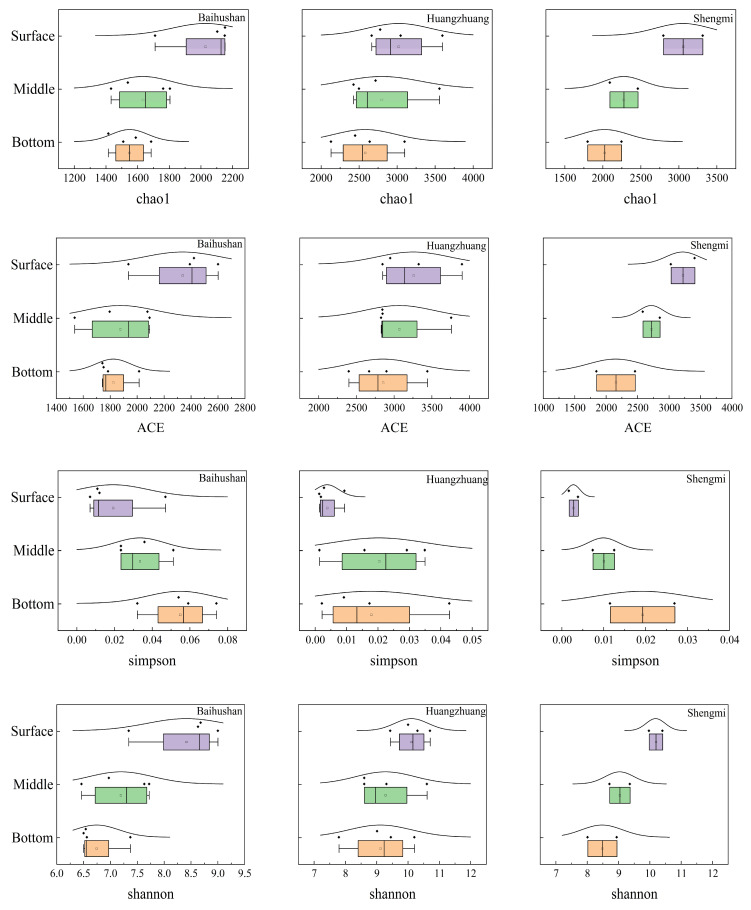
Variation in the α-diversity index. Purple boxplots represent the surface samples, green boxplots represent the middle samples, and orange boxplots represent the bottom samples. Each dot represents a single data point. The curves represent the distribution characteristics of the sample point data.

As shown in Figure 7, according to the statistical analysis, sediment bacterial α-diversity index had a significant trend, and the variation tended to be consistent among the three sample sites. Among of them, Chao1 index, ACE index and Shannon index showed surface > middle > bottom layer. The Simpson index increases gradually with increasing sediment depth. The larger the bacterial Chao1 index and ACE index, the higher the species abundance. This statistic indicates that bacterial abundance in the surface layer of the sediment at the sample site peaked, and that species abundance gradually decreased with increasing depth, probably due to changes in the sediment depositional environment and nutrients. On the other hand, Simpson and Shannon indexes are critical measures of bacterial community diversity. The increase in Simpson index and the decrease in Shannon index from the surface to the bottom layer symbolize the gradual decrease in bacterial diversity in the sample site.

### 3.5. Effects of Environmental Factors on Bacterial Communities

**Figure 8:**
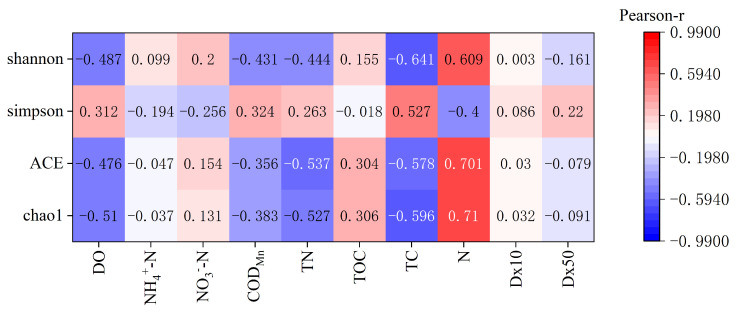
Pearson correlation heat map between environmental factors and bacterial diversity.

As shown in Figure 8 and Table 3, the sample site sediment bacterial Chao1 index showed highly significant negative correlation with interstitial water DO, TN concentration and sediment TC content (*p* < 0.01), significantly negatively correlated with interstitial water COD_Mn_ concentration (*p* < 0.05), and showed a highly significant positive correlation with sediment N content (*p* < 0.01). ACE index showed highly significant negative correlations with interstitial water DO, TN concentration and sediment TC content (*p* < 0.01); There was a highly significant positive correlation with sediment N content (*p* < 0.01). Simpson index showed highly significant positive correlation with sediment TC content (*p* < 0.01), significantly negatively correlated with sediment N content (*p* < 0.05). Shannon index shows highly significant negative correlation with interstitial water DO concentration and sediment N content (*p* < 0.01), significantly negatively correlated with interstitial water COD_Mn_ and TN concentrations (*p* < 0.05), and there was a highly significant positive correlation with sediment N content (*p* < 0.01). In summary, the main environmental factors affecting the abundance and diversity of microorganisms in the sediments of the sample sites in the main tributaries of the Yangtze River were interstitial water DO and TN concentrations and sediment TC and N contents.

**Figure 9:**
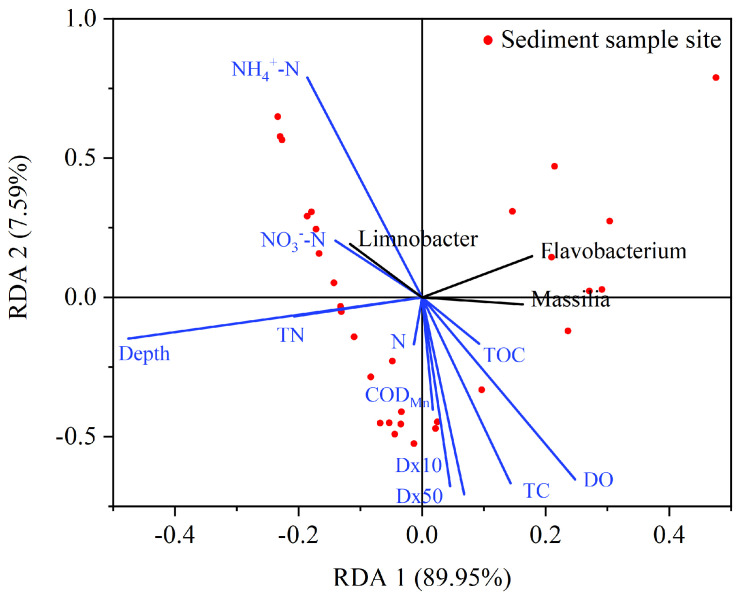
RDA analysis of environmental factors and dominant bacteria at the genus level. Red dots represent 30 sites from three plots Baihushan, Huangzhuang and Shengmi.

Redundancy analysis (RDA) of the top three microorganisms in relative abundance at the genus level at the sample site with environmental factors was performed by using origin 2021b software, and the results are shown in Figure 9. The length of the line segment indicates the strength of the effect of the environmental factor on the bacterial community, with the longer line segment indicating a stronger correlation. Acute angles between line segment connectors indicate a positive correlation, and obtuse angles indicate a negative correlation. The results indicate that the sediment bacterial community at the sample site is strongly influenced by interstitial water DO and NH_4_^+^-N concentrations, sediment TC content and sampling depth. Among them, interstitial water DO and sediment TC were positively correlated with Massilia and Flavobacterium and negatively correlated with Limnobacter. Interstitial water NH_4_^+^-N and sampling depth were positively correlated with Limnobacter and negatively correlated with *Massilia* and *Flavobacterium*. Microorganisms have a certain value of tolerance to environmental factors, and when the environment changes, it causes a modification in the structure of the bacterial community. For instance, the increase in NH_4_^+^-N concentrations due to anthropogenic activities in rivers can inhibit the activity of bacteria associated with autotrophic nitrification in river sediments, leading to a reduction in their abundance and diversity [18].

## 4. Discussion

### 4.1. Patterns of Vertical Distribution of Bacterial Diversity

In the analysis of bacterial community structure in the sandy sediments of the Yangtze River mainstem, results showed no significant differences in the dominant taxa across different sedimentary layers at various sites. This finding contrasts sharply with previous studies and suggests that the vertical distribution pattern of these bacterial communities may be closely linked to the physicochemical characteristics of the sediments and the unique depositional structure of sandy sediments. At the phylum level, *Proteobacteria* dominated across all depths, followed by *Bacteroidetes* and *Acidobacteria.* The extensive distribution and diverse metabolic capabilities of *Proteobacteria* enable them to thrive in the organic matter-rich environments of sediments [19]. In contrast, *Bacteroidetes* and *Acidobacteria* may be less adaptable to these conditions. At the genus level, the dominant species from the surface to the bottom layers were *Massilia*, *Limnobacter* and *Pseudogulbenkiania*. Significant regional specificity was observed among the sample sites, likely influenced by factors such as geographic location, climatic conditions, river characteristics, intensity of human activities, and overall ecosystem health [20]. In many sedimentary environments worldwide, sediment bacterial communities exhibit vertical distribution patterns that vary with depth, typically due to differences in environmental factors such as oxygen concentrations, nutrient availability, and contaminant levels between the surface and bottom layers of the sediment [21,22]. For instance, in a study of bacterial diversity in freshwater lake sediments in Antarctica by Shivaji et al. it was found that the bacterial communities in the surface layer (18–22 cm) and the deeper layer (100–104 cm) were quite different, and that this pattern of distribution depended mainly on oxic-anoxic conditions of the sediment [23].

The results of the bacterial α-diversity index revealed that the diversity was abundant in sediments at different depths, with a notable trend across sedimentary layers. Both the Chao1 and ACE indices decreased with increasing sampling depth, indicating that surface sediments harbored a richer number of species and individuals [24,25]. This is likely due to the direct contact of surface sediments with the water column, which facilitates the influx of organic matter and oxygen, providing a rich source of nutrients and energy for microorganisms. In contrast, the Simpson index was lowest in the surface layer and highest in the bottom layer, suggesting a higher species diversity of bacterial communities in surface sediments [26]. The Shannon index followed a similar pattern, with the highest values in the surface layer and a gradual decrease with depth [27]. This further highlights the high diversity and homogeneity characteristic of surface sediments, whereas bacterial communities in deeper sediments may be dominated by a few species. Similar trends have been observed in other studies, such as those by Ye et al. [28] and Wilms [29], which reported higher bacterial diversity in surface sediments compared to deeper sediments. Notably, Qu et al. [30] found a different pattern in their study of bacterial communities in reservoir sediments, with abundance increasing and then decreasing from the surface to the bottom layer, which may be attributed to the distinct depositional environments of reservoirs and rivers.

The Yangtze River Basin’s unique geography and climate set this study apart from existing research, yielding distinct vertical distribution patterns of sediment microbial communities. The flow of the Yangtze River through diverse climatic zones, ranging from chilly highlands to the warm subtropics, significantly impacts the distribution of sediment microbial communities. The river traverses varied environmental conditions marked by differences in temperature, rainfall and vegetation. These variations not only alter the river’s water chemistry and the nature of its sediments but also play a crucial role in shaping the distribution of sediment bacterial communities along its course [31]. Furthermore, the impact of human activities on bacterial taxa in this region diverges from other studies. The densely populated Yangtze River Basin, with its high levels of industrial and agricultural activity, likely introduces a distinct pattern of pollutant and nutrient inputs to the sediments, differing from streams with minimal human impact. For instance, remote rivers with lower levels of pollution may have sediment bacterial communities more heavily influenced by natural processes [32,33]. Additionally, the sediment transport and deposition processes of the Yangtze River, one of the world’s largest rivers, may differ significantly from those of smaller or slower-flowing rivers. The hydrodynamic properties of rivers, such as flow rate and sediment load, play a crucial role in shaping the distribution and diversity of bacterial communities in sediments [20].

### 4.2. Bacterial Diversity Impact Factor

The sediment samples from the sampling sites were analyzed for physicochemical properties and assessed for bacterial diversity, revealing a clear vertical distribution pattern. As microorganisms are highly sensitive to ecosystem environmental changes, bacterial diversity and community structure characteristics are closely linked to environmental fluctuations. Investigating the impact of environmental factors on bacterial community variability is therefore of great practical significance. For instance, Liu et al. [34] found that bacterial diversity in surface sediments was closely tied to hydrological conditions and organic matter inputs in a different section of the Yangtze River. Similarly, Li et al. [19] highlighted the effect of vertical nutrient distribution in sediments on bacterial community structure. In this study, we selected interstitial water DO, NH_4_^+^-N, NO_3_^−^-N, COD_Mn_, TN, and sediment TOC, TC, N, Dx10, Dx50, and sampling depth as environmental factors for Pearson’s correlation analysis of bacterial alpha diversity indices.

The results showed that both Chao1 and ACE indices were positively correlated with interstitial water DO and TN concentrations and negatively correlated with sediment N content. This suggests that increased DO and TN concentrations can reduce bacterial abundance, consistent with previous findings by Wang et al. [35]. The growth of certain microorganisms may be inhibited under high oxygen conditions, as they may be better adapted to low oxygen or anaerobic environments. The relationship between species abundance and sediment N may reveal a direct effect of organic matter content and nutrient levels on bacterial community structure. High concentrations of organic matter and nutrients are often recognized as key factors promoting bacterial growth and diversity [36,37]. Furthermore, the results of Simpson and Shannon indexes indicate that alterations in interstitial water DO, TN concentration, and sediment N content can also affect bacterial species diversity and homogeneity. Specifically, lower pore water DO and TN concentrations, accompanied by higher sediment N content, correspond to higher species diversity and more uniform distribution.

On the other hand, the dominant bacterial species were analyzed using redundancy analysis (RDA) with respect to environmental factors. The results showed that *Limnobacter* preferred to grow under low oxygen and high nutrient value conditions, while *Massilia* and *Flavobacterium* were more suitable for high oxygen environments. Microorganisms have a certain tolerance to environmental factors, and when the environment changes, it causes a modification in the structure of the bacterial community. For example, variations in NH_4_^+^-N concentration can inhibit the activity of bacteria associated with autotrophic nitrification, leading to a decrease in abundance and diversity [38].

## 5. Conclusions

(1) The abundance and diversity indices of sediment microbes decreased with increasing sediment depth.
(2) The main environmental factors affecting microbial abundance and diversity in the sediments at the sample sites were interstitial water DO, TN concentration and sediment N content.
(3) High oxygen and more nutrient-rich sediment layers provided suitable conditions for *Massilia* and *Flavobacterium* to grow and flourish, while *Limnobacter* preferred low-oxygen or anaerobic conditions.

## Figures and Tables

**Table 1:** Statistical table of the main advantageous microorganisms at the phylum level.

Sample	Depths	Bacterial
Baihushan	Surface	*Proteobacteria*, *Bacteroidetes*
	Middle	*Proteobacteria*, *Bacteroidetes*
	Bottom	*Proteobacteria*, *Bacteroidetes*
Huangzhuang	Surface	*Proteobacteria*, *Acidobacteria*
	Middle	*Proteobacteria*, *Acidobacteria*
	Bottom	*Proteobacteria*, *Acidobacteria*
Shengmi	Surface	*Proteobacteria*, *Acidobacteria*
	Middle	*Proteobacteria*, *Acidobacteria*
	Bottom	*Proteobacteria*, *Acidobacteria*

**Table 2:** Statistical table of the main advantageous bacterial at the genus level.

Sample	Depths	Bacterial
Baihushan	Surface	*Massilia*
	Middle	*Massilia*
	Bottom	*Flavobacterium*
Huangzhuang	Surface	*Limnobacter*
	Middle	*Limnobacter*
	Bottom	*Limnobacter*
Shengmi	Surface	*Pseudogulbenki*
	Middle	*Pseudogulbenki*
	Bottom	*Pseudogulbenki*

**Table 3:** Pearson correlation coefficient between environmental factors and bacterial diversity.

Physicochemical Indicators	Chao1	ACE	Simpson	Shannon
DO	−0.510 **	−0.476 **	0.312	−0.487 **
NH_4_^+^-N	−0.037	−0.047	−0.194	0.099
NO_3_^−^-N	0.131	0.154	−0.256	0.200
COD_Mn_	−0.383 *	−0.356	0.324	−0.431 *
TN	−0.527 **	−0.537 **	0.263	−0.444 *
TOC	0.306	0.304	−0.018	0.155
TC	−0.596 **	−0.578 **	0.527 **	−0.641 **
N	0.710 **	0.701 **	−0.400 *	0.609 **
Dx10	0.032	0.030	0.086	0.003
Dx50	−0.091	−0.079	0.220	−0.161

Note: * *p* < 0.05; ** *p* < 0.01.

## Data Availability

The data presented in this study are available on request.

## References

[1] Yang Q.H., Zhou H.Y., Ji F.W., Wang H., Yang W.F. (2008). Bioturbation in Seabed Sediments and Its Effects on Marine Sedimentary Processes and Records. Adv. Earth Sci..

[2] Kostka J.E. (2013). Controls of the microbial nitrogen cycle in marine sediments and implications for global climate change. Abstr. Pap. Am. Chem. Soc..

[3] Linn J.H., Eike B., Philip W.B., Keith A.H. (2012). Influence of ocean warming and acidification on trace metal biogeochemistry. Mar. Ecol. Prog. Ser..

[4] Allen J.G., Beutel M.W., Call D.R., Fischer A.M. (2010). Effects of oxygenation on ammonia oxidation potential and microbial diversity in sediment from surface-flow wetland mesocosms. Bioresour. Technol..

[5] Wyness A.J., Fortune I., Blight A.J., Browne P., Hartley M., Holden M., Paterson D.M. (2021). Ecosystem engineers drive differing microbial community composition in intertidal estuarine sediments. PLoS ONE.

[6] Liang S., Li H., Wu H., Yan B., Song A. (2023). Microorganisms in coastal wetland sediments: A review on microbial community structure, functional gene, and environmental potential. Front. Microbiol..

[7] Hugh F., Peter T., Gary A.K. (2013). Shifts in composition of microbial communities of subtidal sandy sediments maximise retention of nutrients. FEMS Microbiol. Ecol..

[8] Probandt D., Eickhorst T., Ellrott A., Amann R., Knittel K. (2017). Microbial life on a sand grain: From bulk sediment to single grains. ISME J..

[9] Dighton J., White J.F. (2017). The Ecology of Fungi: An Environmental Perspective.

[10] Sun Y.M., Tang K.X., Ma Y., Zhu X., Li H.Y., Zhang F., Chen S., Huang H.P. (2022). Variations in nutrients and microbes during the occurrence and extinction of algal blooms: A mesocosm experiment with the addition of marine aquaculture sediment. Front. Mar. Sci..

[11] Hong Y.G., Wu J.P., Wilson S., Song B.K. (2019). Vertical Stratification of Sediment Microbial Communities Along Geochemical Gradients of a Subterranean Estuary Located at the Gloucester Beach of Virginia, United States. Front. Microbiol..

[12] Guo X.P., Yang Y., Niu Z.S., Lu D.P., Zhu C.H., Feng J.N., Wu J.Y. (2019). Characteristics of microbial community indicate anthropogenic impact on the sediments along the Yangtze Estuary and its coastal area. Sci. Total Environ..

[13] Liu S., Ren H.X., Shen L.D., Lou L.P., Tian G.M., Zheng P., Hu B.L. (2015). PH levels drive bacterial community structure in sediments of the Qiantang Riveras determined by 454 pyrosequencing. Front. Microbiol..

[14] Liu N., Wang B.L., Yang M.L., Li W.Z., Shi X.J., Liu C.Q. (2023). The different responses of planktonic bacteria and archaea to water temperature maintain the stability of their community diversity in dammed rivers. Ecol. Process..

[15] Tang X.Q., Li R., Han D., Scholz M. (2020). Response of Eutrophication Development to Variations in Nutrients and Hydrological Regime: A Case Study in the Changjiang River (Yangtze) Basin. Water.

[16] State Environmental Protection Administration (2002). Water and Wastewater Monitoring and Analysis Methods.

[17] Li S.J., Xu C.B., Qing S., Guo X., Bai Y.C., Guo F. (2022). Molecular characteristics of biochar-derived organic matter sub-fractions extracted by ultrasonication. Sci. Total Environ..

[18] Guan Y., Hou T., Li X., Feng L., Wang Z. (2022). Metagenomic insights into comparative study of nitrogen metabolic potential and microbial community between primitive and urban river sediments. Environ. Res..

[19] Li Y., Fan L.H., Zhang W.L. (2020). How did the bacterial community respond to the level of urbanization along the Yangtze River?. Environ. Sci. Process. Impacts.

[20] Wang L., Zhang J., Li H., Yang H., Peng C., Peng Z., Lu L. (2018). Shift in the microbial community composition of surface water and sediment along an urban river. Sci. Total Environ..

[21] Zhang Y.H., Yao P., Sun C., Li S.Z., Shi X.C., Zhang X.H., Liu J.W. (2021). Vertical diversity and association pattern of total, abundant and rare microbial communities in deep-sea sediments. Mol. Ecol..

[22] Wurzbacher C., Fuchs A., Attermeyer A., Frindte K., Grossart H.P., Hupfer M., Casper P., Monaghan M.T. (2017). Shifts among Eukaryota, Bacteria, and Archaea define the vertical organization of a lake sediment. Microbiome.

[23] Shivaji S., Kumari K., Kishore K.H., Pindi P.K., Rao P.S., Srinivas T.R.N., Asthana R., Ravindra R. (2011). Vertical distribution of bacteria in a lake sediment from Antarctica by culture-independent and culture-dependent approaches. Res. Microbiol..

[24] Wang C., Liu D.W., Bai E. (2018). Decreasing soil microbial diversity is associated with decreasing microbial biomass under nitrogen addition. Soil. Biol. Biochem..

[25] Chernov T.I., Tkhakakhova A.K., Kutovaya O.V. (2015). Assessment of Diversity Indices for the Characterization of the Soil Prokaryotic Community by Metagenomic Analysis. Eurasian Soil. Sci..

[26] Louis B.P., Maron P.A., Menasseri-Aubry S., Sarr A., Lévêque A., Mathieu O., Jolivet C., Leterme P., Viaud V. (2016). Microbial Diversity Indexes Can Explain Soil Carbon Dynamics as a Function of Carbon Source. PLoS ONE.

[27] Wagner B.D., Grunwald G.K., Zerbe G.O., Mikulich-Gilbertson S.K., Robertson C.E., Zemanick E.T., Harris J.K. (2018). On the use of diversity measures in longitudinal sequencing studies of microbial communities. Front. Microbiol..

[28] Ye Q., Wu Y., Zhu Z.Y., Wang X.N., Li Z.Q., Zhang J. (2016). Bacterial diversity in the surface sediments of the hypoxic zone near the Changjiang Estuary and in the East China Sea. MicrobiologyOpen.

[29] Wilms R., Köpke B., Sass H., Chang T.S., Cypionka H., Engelen B.J.E.M. (2006). Deep biosphere-related bacteria within the subsurface of tidal flat sediments. Environ. Microbiol..

[30] Qu J.H., Yuan H.L., Huang H.Z., Wang E.T. (2005). Characteristics of the longitudinal distribution of bacterial communities in the Guanting Reservoir sediment. Sci. China Ser. D-Earth Sci..

[31] Liu T., Zhang A.N., Wang J.W., Liu S.F., Jiang X.T., Dang C.Y., Ma T., Liu S.T. (2018). Integrated biogeography of planktonic and sedimentary bacterial communities in the Yangtze River. Microbiome.

[32] Allan J.D., Castillo K.A. (2007). Stream Ecology: Structure and Function of Running Waters.

[33] Allan J.D., Castillo M.M., Capps K.A. (2021). Stream Ecology: Structure and Function of Running Waters.

[34] Liu W., Zhang D., Wang S., Zhao J., Yao H.Y. (2022). Risk assessments of emerging contaminants in various waters and changes of microbial diversity in sediments from Yangtze River chemical contiguous zone, Eastern China. Sci. Total Environ..

[35] Wang J., Shan S., Li D., Zhang Z., Ma Q. (2023). Long-term influence of chloroxylenol on anaerobic microbial community: Performance, microbial interaction, and antibiotic resistance gene behaviors. Sci. Total Environ..

[36] Traving S.J., Rowe O., Jakobsen N.M., Sørensen H., Dinasquet J., Stedmon C.A., Andersson A., Riemann L. (2017). The effect of increased loads of dissolved organic matter on estuarine microbial community composition and function. Front. Microbiol..

[37] Freixa A., Ejarque E., Crognale S., Amalfitano S., Fazi S., Butturini A., Romanı A.M. (2016). Sediment microbial communities rely on different dissolved organic matter sources along a Mediterranean river continuum. Limnol. Oceanogr..

[38] Wang K., Ke S.Z., Yuan H.Z., Zhu J., Li J.W. (2020). Effect of ammonia-nitrogen concentration on bacterial community structure in a MBBR process. Environ. Eng..

